# t(8;21)急性髓系白血病异基因造血干细胞移植后复发的危险因素分析

**DOI:** 10.3760/cma.j.issn.0253-2727.2021.12.006

**Published:** 2021-12

**Authors:** 雯雯 郭, 欣 刘, 爱明 庞, 卫华 翟, 栋林 杨, 欣 陈, 巧玲 马, 祎 何, 荣莉 张, 四洲 冯, 明哲 韩, 尔烈 姜

**Affiliations:** 中国医学科学院血液病医院（中国医学科学院血液学研究所），实验血液学国家重点实验室，国家血液系统疾病临床医学研究中心，细胞生态海河实验室天津 300020 State Key Laboratory of Experimental Hematology, National Clinical Research Center for Blood Diseases, Haihe Laboratory of Cell Ecosystem, Institute of Hematology & Blood Diseases Hospital, Chinese Academy of Medical Sciences & Peking Union Medical College, Tianjin 300020, China

**Keywords:** 造血干细胞移植, t(8;21)急性髓系白血病, 微小残留病, 复发, Hematopoietic stem cell transplantation, Leukemia, myeloid, acute, t(8;21), Minimal residual diseases, Relapse

## Abstract

**目的:**

探讨t(8;21)急性髓系白血病（AML）异基因造血干细胞移植（allo-HSCT）后复发的影响因素。

**方法:**

对2008年1月至2020年10月期间在中国医学科学院血液病医院接受allo-HSCT的t(8;21) AML患者进行回顾性分析。

**结果:**

73例t(8;21) AML患者纳入研究，男39例，女34例，中位年龄36（13～54）岁。73例患者中10例出现血液学复发，3年累积复发率（CIR）为15.7％（95％*CI* 7.3％～26.8％），中位复发时间为9.2（2.0～47.6）个月。73例患者中19例死亡，移植后3年总生存（OS）率为68.9％（95％*CI* 56.4％～81.4％）。移植后3个月内融合基因下降≥3个对数级组移植后3年CIR明显低于下降<3个对数级组［13.3％（95％*CI* 4.5％～26.8％）对57.1％（95％*CI* 13.2％～85.6％），*P*<0.001］；移植后12个月内融合基因水平下降≥4个对数级组移植后3年CIR明显低于下降<4个对数级组［5.1％（95％*CI* 0.9％～15.4％）对25.0％（95％*CI* 0.3％～71.3％），*P*<0.001］。Cox多因素分析显示移植当天造血干细胞回输前（0 d）RUNX1-RUNX1T1融合基因高定量（≥1.58％）［*P*＝0.006，*HR*＝28.849（95％*CI* 2.680～310.524）］及初诊时骨髓流式细胞术原始细胞比例≥60％［*P*＝0.015，*HR*＝6.640（95％*CI* 1.448～30.457）］是血液学复发的独立危险因素；c-Kit及Flt3基因突变对移植后血液学复发无明显影响（*P*＝0.877，*P*＝0.773）。初诊时骨髓流式细胞术原始细胞比例≥60％［*P*<0.001，*HR*＝8.925（95％*CI* 2.702～29.476）］、达到完全缓解所需的疗程数≥2个［*P*＝0.013，*HR*＝4.495（95％*CI* 1.379～14.649）］是影响OS的独立危险因素。

**结论:**

0 d RUNX1-RUNX1T1融合基因定量水平≥1.58％及初诊时骨髓流式细胞术原始细胞比例≥60％是影响t(8;21) AML患者allo-HSCT后血液学复发的独立危险因素。移植后RUNX1-RUNX1T1融合基因监测有助于评估复发的危险性。

## Abstract (en)

**Objective:**

To investigate the risk factors of relapse after allogeneic hematopoietic stem cell transplantation（allo-HSCT）in patients with t(8;21) acute myeloid leukemia (AML).

**Methods:**

The clinical features of patients with t(8;21) AML who received allo-HSCT between January 2008 and October 2020 in the Hospital of Blood Disease and the Chinese Academy of Medical Sciences were retrospectively analyzed. Univariate and multivariate analyses were performed on the factors that might influence relapse.

**Results:**

A total of 73 patients were enrolled. The analysis revealed that, out of the 73 cases, 10 had relapses, with a 3-year cumulative incidence of relapse (CIR) of 15.7％ (95％ *CI* 7.3％–26.8％). The median time of relapse was 9.2 (2.0–47.6) months. Furthermore, 19 cases died, with a 3-year overall survival (OS) of 68.9％ (95％ *CI* 56.4％–81.4％). Compared with the RUNX1-RUNX1T1 at first diagnosis, a ≥ 3-log reduction within 3 months and/or 4-log reduction within 3-12 months can significantly decrease 3-year CIR after HSCT (13.3％ *vs* 57.1％; 5.1％ *vs* 25.0％, both *P*<0.001). Cox multivariate analysis showed that high levels of RUNX1-RUNX1T1 (≥1.58％) on the day of transplantation (day 0) [*P*＝0.006; *HR*＝28.849 (95％ *CI* 2.68–310.524)] and the flow cytometric analysis of blasts ratio in bone marrow ≥60％ at first diagnosis [*P*＝0.015; *HR*＝6.64 (95％ *CI* 1.448–30.457)] were independent risk factors for relapse. Furthermore, no significant difference in the effect of c-Kit and Flt3 gene mutations on relapse after transplantation was observed (*P*＝0.877 and *P*＝0.773, resp). The flow cytometric analysis of blasts ratio in bone marrow ≥60％ at first diagnosis [*P*<0.001; *HR*＝8.925 (95％ *CI* 2.702–29.476)] and the number of courses to achieve complete remission ≥ 2 [*P*＝0.013; *HR*＝4.495 (95％ *CI* 1.379–14.649)] were independent risk factors for OS.

**Conclusion:**

Both high levels of RUNX1-RUNX1T1 (≥1.58％) on the day of transplantation (day 0) and the ratio of flow cytometric analysis of blasts in bone marrow at first diagnosis increase the chance of t(8;21) AML relapse after allo-HSCT. Detection of the transcription levels of RUNX1-RUNX1T1 after allo-HSCT at different times could help predict the hazard of relapse.

t(8;21)急性髓系白血病（AML）是急性白血病的一种特殊亚类，存在由t(8;21)产生的RUNX1-RUNX1T1融合基因是这类白血病的分子学特征，由于对化疗的敏感性高，通常被认为预后良好[1]–[2]。但是仍有约40％的t(8;21) AML患者出现血液学复发，长期生存率不足50％，其中合并c-Kit基因突变患者的复发率可达70％且预后不良[1],[3]–[4]。异基因造血干细胞移植（allo-HSCT）是唯一具有治愈潜力的手段。同时血液学复发也是造成t(8;21) AML患者allo-HSCT治疗失败的主要原因[4]–[5]。目前尚缺乏有效的识别移植后高危复发人群的指标。本研究对73例行allo-HSCT的t(8;21) AML患者进行回顾性分析，探究影响移植后血液学复发的危险因素。

## 病例与方法

1. 病例资料：2008年至2020年10月在我科接受治疗的t(8;21) AML患者共119例，其中接受allo-HSCT、移植前达到完全缓解（CR）且数据资料完整的有73例，以73例t(8;21) AML患者为研究对象，收集资料主要包括年龄、性别等一般情况，初诊时血常规、骨髓形态学及流式细胞术检测原始细胞比例、融合基因定性定量、染色体核型异常以及化疗疗效等情况；移植后定期检测融合基因以及分子生物学，随访血液学复发、死亡等预后情况。

2. 定义：t(8;21) AML的诊断标准：骨髓或外周血原始细胞比例≥20％；伴有t(8;21)(q22; q22)染色体异常；RUNX1-RUNX1T1融合基因阳性。如果染色体分裂象少或失败则必须满足RUNX1-RUNX1T1融合基因阳性，如果存在t(8;21)或RUNX1-RUNX1T1异常时，骨髓原始细胞即使<20％也可诊断t(8;21) AML。分子生物学阳性包括分子生物学复发及分子生物学持续阳性，前者指骨髓样本RQ-PCR定量检测RUNX1-RUNX1T1融合基因由阴性转为阳性，或者定性检测由阴性转为阳性；后者指的是连续3次及以上RUNX1-RUNX1T1融合基因定性或者定量检测为阳性，两者均未达到形态学复发标准且无髓外复发。血液学复发定义为RUNX1-RUNX1T1融合基因转为阳性，并经复查证实骨髓形态学复发或者发生髓外白血病。

3. 移植前状况：移植前所有患者均接受诱导化疗，CR后巩固治疗至少2疗程；均在移植前获得CR，其中处于第1、2次CR（CR1、CR2）者分别为60、13例。患者接受allo-HSCT的指征：①巩固化疗后分子生物学持续阳性或早期复发趋势；②出现血液学复发；③预后不良的基因突变，如c-Kit基因突变（包括外显子17和外显子8突变）；④患者意愿。

4. 供者、干细胞来源及移植类型：亲缘供者66例，无关供者7例；供受者HLA全相合者45例，其中亲缘全相合40例，无关全相合5例；供受者HLA不全相合28例，亲缘供者不相合26例，无关供者不相合2例，包括2例10位点不全相合（9/10、8/10）。外周血干细胞移植72例，骨髓+外周血干细胞移植1例。单倍型移植26例，同胞全相合供者移植40例，无关供者移植7例。

5. 预处理方案：所有患者均接受清髓性预处理。

6. 融合基因RUNX1-RUNX1T1检测：融合基因RUNX1-RUNX1T1定量检测采用RQ-PCR法，内参基因选用ABL基因，融合基因转录水平等于融合基因转录子拷贝数/ABL转录子拷贝数×100％。在预处理前、移植当天造血干细胞回输前（0 d）、移植后14 d、28 d、42 d、56 d、3个月、6个月、9个月及12个月检测融合基因RUNX1-RUNX1T1定量和（或）定性。

在我科接受治疗的48例t(8;21) AML患者初诊时中位融合基因定量是220％（12％～1393％）。RQ-PCR法对目的基因的检测灵敏度是10拷贝/反应体系，确保未检测到融合基因拷贝数的样本中，内参基因拷贝数大于5×10^4^，因此与初诊时融合基因水平相比，至少能检测到4个对数级水平的下降。移植后（不包括移植当天）在规定时间内收集的样本数据完整的患者占71.2％（52/73），21例数据不完整患者中，缺失1个时间点数据7例，缺失2个时间点数据6例，8例缺失时间点在3个及以上。共收集骨髓样本501份，占试验设计要求的86％。

7. 随访：采用门诊和电话随访。随访截止日期为2021年2月28日。中位随访时间为24.1（12.3～35.9）个月，73例患者中5例失访。主要观察指标：血液学复发时间（从造血干细胞回输到骨髓白血病细胞≥5％或发生髓外复发的时间间隔）；次要观察指标：总生存（OS）时间（从造血干细胞回输至死亡或末次随访的时间间隔）。

8. 统计学处理：采用SPSS 26.0及R 4.0.4软件进行数据分析。累积复发率（CIR）采用竞争风险模型分析，Gray检验比较组间差异；OS采用Kaplan-Meier曲线进行描述，组间比较采用Log-rank检验；将单因素分析*P*<0.3的因素纳入Cox回归模型进行多因素分析。分类变量采用卡方检验。双侧检验*P*<0.05为差异有统计学意义。

## 结果

1. 患者基本资料：本研究纳入73例接受allo-HSCT的t(8;21) AML患者，男39例，女34例，中位年龄36（13～54）岁，其中>35岁者37例，≤35岁者36例。临床资料详见表1。

2. 复发及危险因素分析：73例患者中有10例发生血液学复发，中位复发时间为移植后9.2（2.0～47.6）个月，其中8例因复发死亡，中位死亡时间为移植后2.73（0.07～11.17）个月。移植后3年CIR为15.7％（7.3％～26.8％）。复发曲线见图1。

利用ROC曲线获得预处理前与移植当天干细胞输注前（0 d）融合基因定量阈值。预处理前融合基因定量的ROC曲线面积是0.731（95％*CI* 0.564～0.897）（*P*＝0.021）；最佳截断点是0.0006（0.06％）；0 d融合基因定量的ROC曲线面积为0.763（95％*CI*0.601～0.924）（*P*＝0.017），最佳截断点是0.0158（1.58％），因此我们将≥1.58％、<1.58％定义为0 d融合基因高定量、低定量。

**表1:** 73例接受异基因造血干细胞移植t(8;21)急性髓系白血病患者的一般资料

一般特点	结果
性别［例（％）］	
男	39（53.4）
女	34（46.6）
移植年龄［岁，*M*（范围）］	36（13～54）
初诊WBC［×10^9^/L，*M*（范围）］	11.1（4.1～19.5）
初诊骨髓形态学原始细胞［例（％）］	
≥60％	30（41.1）
<60％	33（45.2）
缺失	10（13.7）
初诊骨髓流式细胞术原始细胞比例［例（％）］	
≥60％	15（20.5）
<60％	47（64.4）
缺失	11（15.1）
染色体核型分析［例（％）］	
单纯t(8;21)	35（47.9）
性染色体异常	29（39.7）
其他	9（12.3）
c-Kit基因［例（％）］	
野生型	30（41.1）
突变	25（34.2）
未知	18（24.7）
FLT3基因［例（％）］	
野生型	56（76.7）
突变型	9（12.3）
未知	8（11.0）
达到CR的疗程数［例（％）］	
1个	50（68.5）
≥2个	23（31.6）
移植前状态［例（％）］	
CR1	60（82.2）
≥CR2	13（17.8）
移植类型［例（％）］	
单倍型	26（35.6）
同胞全相合	40（54.8）
无关供者	7（9.6）
干细胞来源［例（％）］	
外周血干细胞	72（98.6）
骨髓+外周血干细胞	1（1.4）
预处理前融合基因［例（％）］	
高定量（≥0.06％）	24（32.9）
低定量（<0.06％）	49（67.1）
0 d融合基因［例（％）］	
高定量（≥1.58％）	23（31.5）
低定量（<1.58％）	50（68.5）
移植后6个月内分子生物学检测结果［例（％）］	
阴性	39（53.4）
持续阳性	12（16.4）
分子生物学复发	22（30.1）

注：CR：完全缓解；CR1、CR2分别为第1、2次CR；0 d：移植当天造血干细胞回输前

**图1:**
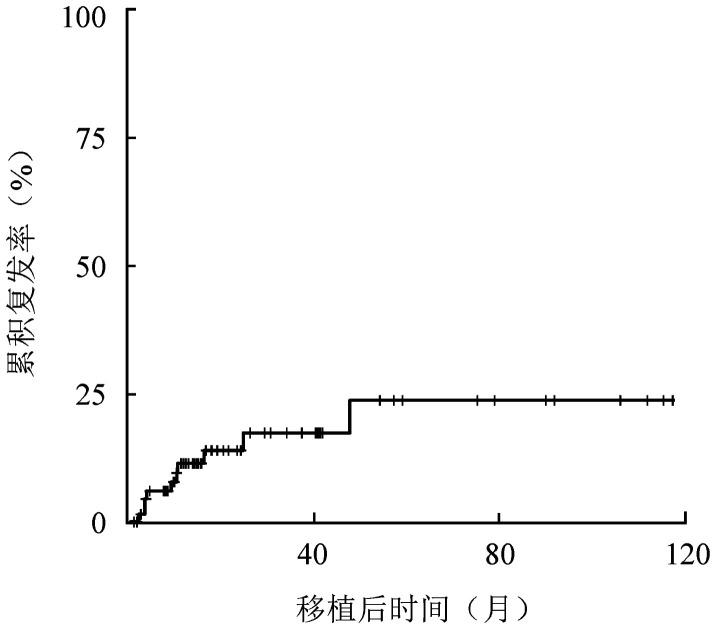
73例t(8;21)急性髓系白血病患者异基因造血干细胞移植后复发曲线

对可能影响复发的因素进行分析，如表2所示。预处理前融合基因高定量组24例，低定量组49例，0 d融合基因高定量23例，低定量50例。预处理高、低定量组移植后3年CIR分别为44.1％（95％*CI* 17.2％～68.3％）、2.5％（95％*CI* 0.2％～11.3％）（*P*<0.001）。0 d融合基因高、低定量组移植后3年CIR分别为42.8％（95％*CI* 16.8％～66.8％）、2.6％（95％*CI* 0.2％～11.7％）（*P*<0.001）（图2）。

**表2:** 影响t(8;21)急性髓系白血病患者allo-HSCT后血液学复发的单因素分析

因素	3年CIR［％，*M*（95％*CI*）］	统计量	*P*值
性别		0.090	0.765
男	17.1（5.7～35.1）		
女	13.4（4.1～28.3）		
年龄		1.885	0.170
>35岁	10.4（2.4～25.3）		
≤35岁	21.2（8.0～38.4）		
初诊WBC		0.180	0.672
≥20×10^9^/L	22.6（4.5～48.9）		
<20×10^9^/L	12.9（5.0～24.4）		
初诊骨髓形态学原始细胞比例		1.277	0.259
≥60％	29.3（9.3～53.1）		
<60％	11.5（2.7～27.4）		
初诊骨髓流式细胞术原始细胞比例		7.027	0.008
≥60％	42.3（12.5～70.1）		
<60％	5.9（1.0～17.5）		
染色体核型		0.343	0.558
单纯t(8;21)	10.8（2.6～25.7）		
其他	18.5（7.1～34.0）		
c-kit基因		0.024	0.877
突变	22.1（5.0～46.6）		
野生型	19.8（6.9～37.6）		
Flt3基因		0.083	0.773
突变	15.2（0.4～52.3）		
野生型	18.9（8.2～32.9）		
移植类型		0.183	0.669
单倍型	16.1（3.5～37.2）		
同胞全相合	18.3（7.1～33.7）		
移植前达CR疗程数		2.978	0.084
1个	10.3（3.0～22.9）		
≥2个	27.3（8.8～50.1）		
移植前状态		0.141	0.707
CR1	18.6（7.9～32.8）		
≥CR2	7.7（0.4～30.4）		
预处理前融合基因定量		12.269	<0.001
高定量（≥0.06％）	44.1（17.2～68.3）		
低定量（<0.06％）	2.5（0.2～11.3）		
0 d融合基因定量		13.170	<0.001
高定量（≥1.58％）	42.8（16.8～66.8）		
低定量（<1.58％）	2.6（0.2～11.7）		
移植后6个月内发生分子生物学阳性		6.739	0.009
是	30.5（12.3～51.0）		
否	3.2（0.2～14.3）		

注：CR：完全缓解；CR1、CR2分别为第1、2次CR；0 d：移植当天造血干细胞回输前

**图2:**
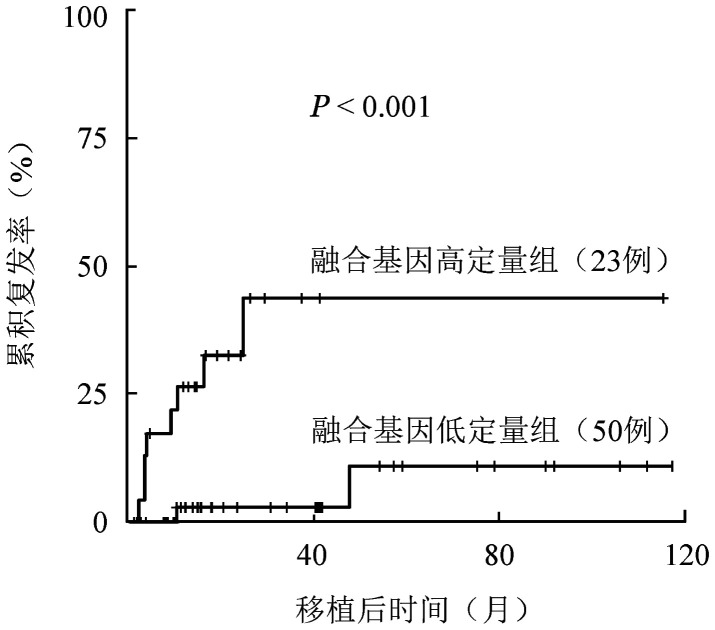
移植当天干细胞回输前融合基因高定量（≥1.58％）和低定量（<1.58％）t(8;21)急性髓系白血病患者异基因造血干细胞移植后复发曲线

通过对移植后融合基因的定量检测，我们发现与初诊时融合基因水平相比，移植后1个月内融合基因下降水平是否≥3个对数级对移植后3年CIR无明显影响，其中≥3个、<3个对数级组移植后3年CIR分别为16.4％（95％*CI* 7.1％～28.9％）、25.0％（95％*CI* 0.3％～71.4％）（*P*＝0.559）；但是移植后1～3个月内融合基因下降水平≥3个对数级组移植后3年CIR明显低于<3个对数级组［13.3％（95％*CI* 4.5％～26.8％）对57.1％（95％*CI* 13.2％～85.6％），*P*<0.001］；在移植后12个月时融合基因水平下降≥4个对数级组移植后3年CIR明显低于<4个对数级者［5.1％（95％*CI* 0.9％～15.4％）对25.0％（95％*CI* 0.3％～71.3％），*P*<0.001］。

初诊时骨髓流式细胞术原始细胞≥60％、<60％组的移植后3年CIR分别为42.3％（95％*CI* 12.5％～70.1％）、5.9％（95％*CI* 1.0％～17.5％）（*P*＝0.008）。移植后6个月内分子生物学阳性、阴性分别为34、39例，移植后3年CIR分别为30.5％（95％*CI* 12.3％～51.0％）、3.2％（95％*CI* 0.2％～14.3％）（*P*＝0.009）。而初诊时骨髓形态学原始细胞比例≥60％、<60％组的移植后3年CIR分别为29.3％（95％*CI* 9.3％～53.1％）、11.5％（95％*CI*2.7％～27.4％）（*P*＝0.259）。c-kit基因突变组、野生型组移植后3年CIR分别为22.1％（95％*CI* 5.0％～46.6％）、19.8％（95％*CI* 6.9％～37.6％）（*P*＝0.877）。单倍型移植与同胞全相合移植3年CIR分别为16.1％（95％*CI* 3.5％～37.2％）、18.3％（95％*CI* 7.1％～33.7％）（*P*＝0.669）。

3. 生存情况：中位随访24.1（12.3～35.9）个月，共有49例存活，其中48例为无病生存；5例失访；19例死亡，死于血液学复发者8例，非复发死亡者11例，包括死于重症感染者或重度GVHD者9例，其他2例。移植后3年的OS率为68.9％（95％*CI* 56.4％～81.4％），OS曲线见图3。单因素分析发现，初诊时形态学骨髓原始细胞比例≥60％、<60％组移植后3年OS率分别为57.8％（95％*CI* 34.1％～81.5％）、84.2％（95％*CI* 69.3％～99.1％）（*P*＝0.031）；初诊时骨髓流式细胞术原始细胞比例≥60％、<60％组的移植后3年OS率分别为34.6％（95％*CI* 6.4％～68.8％）、86.0％（95％*CI* 74.2％～97.8％）（*P*<0.001）。达到CR所需的疗程数为1、≥2两组3年OS率分别为78.3％（95％*CI* 65.4％～91.2％）、42.2％（95％*CI* 15.3％～69.1％）（*P*＝0.017）。详见表3。

**图3:**
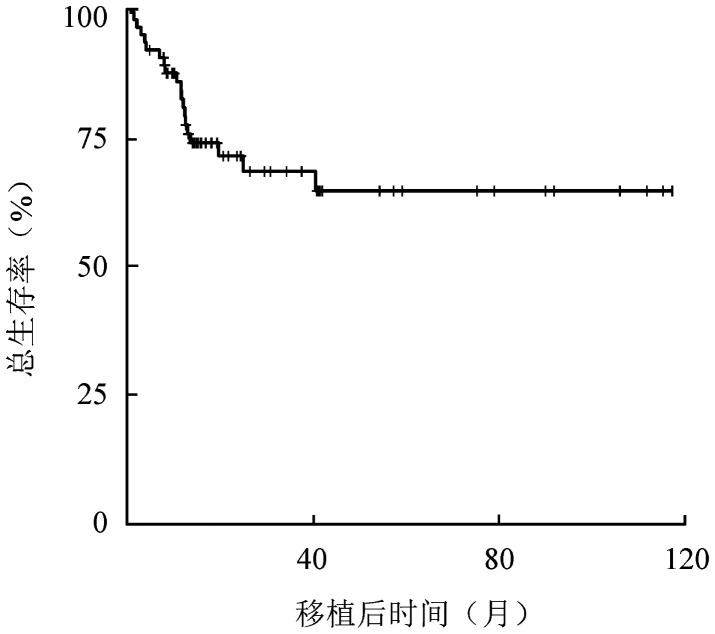
73例t(8;21)急性髓系白血病患者异基因造血干细胞移植后总生存曲线

**表3:** 影响t(8;21)急性髓系白血病患者allo-HSCT后总生存（OS）的单因素分析

因素	3年OS率［％，*M*（95％*CI*）］	*χ*^2^值	*P*值
性别		0.020	0.888
男	63.9（45.7~82.1）		
女	75.3（59.2~91.4）		
年龄		0.012	0.913
>35岁	70.3（53.2~87.3）		
≤35岁	67.1（48.9~85.3）		
初诊外周血白细胞计数		0.696	0.404
≥20×10^9^/L	57.7（31.0~84.3）		
<20×10^9^/L	73.4（60.1~86.7）		
初诊骨髓形态学原始细胞比例		4.630	0.031
≥60％	57.8（34.1～81.5）		
<60％	84.2（69.3~99.1）		
初诊流式细胞术骨髓原始细胞比例		18.197	<0.001
≥60％	34.6（6.4~68.8）		
<60％	86.0（74.2~97.8）		
染色体核型		1.718	0.190
单纯t(8;21)	79.8（65.3~94.3）		
其他	61.6（43.0~80.2）		
c-kit基因		0.103	0.749
突变型	66.5（41.4~91.6）		
野生型	72.3（54.1~90.5）		
Flt3基因		0.596	0.440
突变型	61.0（25.5~96.5）		
野生型	68.7（53.6~83.8）		
移植类型		0.006	0.940
单倍型	66.7（44.0~89.4）		
同胞全相合	69.2（53.7~84.7）		
达到CR的疗程数		5.660	0.017
1个	78.3（65.4~91.2）		
≥2个	42.2（15.3~69.1）		
移植前状态		0.014	0.904
CR1	66.2（50.9~81.5）		
≥CR2	75（50.5~99.5）		
预处理前融合基因		1.723	0.189
高定量（≥0.06％）	51.1（24.1~78.1）		
低定量（<0.06％）	76.0（63.1~88.9）		
0 d融合基因		0.626	0.429
高定量（≥1.58％）	53（26.3~79.7）		
低定量（<1.58％）	76（62.9~89.1）		
移植后6月内出现分子生物学阳性		0.352	0.553
是	64.1（43.1~85.1）		
否	71.9（56.0~87.8）		

注：CR：完全缓解；CR1、CR2分别为第1、2次CR；0 d：移植当天造血干细胞回输前

4. 多因素分析：将单因素分析*P*值<0.3的纳入多因素分析，通过Cox多因素回归分析发现：0 d融合基因高定量及初诊时骨髓流式细胞术原始细胞比例≥60％是移植后复发的独立危险因素。初诊时骨髓流式细胞术原始细胞比例≥60％，达到完全缓解所需的疗程数≥2是OS的独立危险因素（表4）。根据造成移植后血液学复发的危险因素（初诊时骨髓流式细胞术原始细胞比例≥60％、0 d融合基因高定量）分为高危组（具备2个危险因素，7例）、中危组（具备1个危险因素，24例）和低危组（不具备危险因素，42例），移植后3年CIR分别为57.1％、14.8％、3.0％（*P*<0.001）（图4）。根据影响OS的危险因素分为高危组（具备2个危险因素，5例）、中危组（具备1个危险因素，28例）和低危组（不具备危险因素，40例），移植后3年OS率分别为0％、52.5％、86.1％（*P*<0.001）。

**表4:** t(8;21)急性髓系白血病患者allo-HSCT后累积复发率（CIR）及总生存（OS）影响因素的多因素分析

影响因素	*HR*（95%*CI*）	*P*值
CIR		
0 d融合基因高定量（≥1.58%）	28.849（2.680~310.524）	0.006
初诊骨髓流式细胞术原始细胞比例≥60%	6.640（1.448~30.457）	0.015
OS		
达到完全缓解所需的疗程数≥2	4.495（1.379~14.649）	0.013
初诊骨髓流式细胞术原始细胞比例≥60%	8.925（2.702~29.476）	<0.001

注：0 d：移植当天造血干细胞回输前

**图4:**
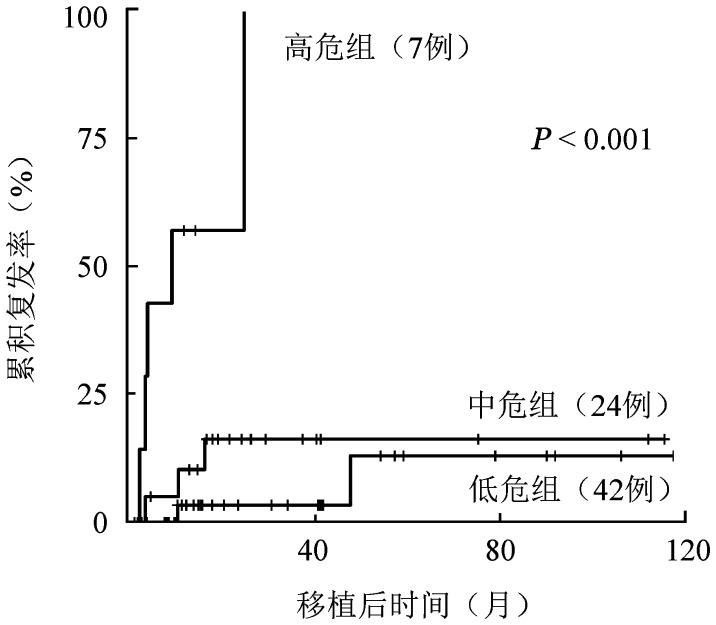
不同复发危险度分组t(8;21)急性髓系白血病患者异基因造血干细胞移植后复发曲线

## 讨论

复发是t（8；21）AML治疗失败的主要原因[1],[6]–[7]。近年来，对具有高危复发风险的t(8;21) AML患者行allo-HSCT被认为是降低复发率、改善预后的重要方法[3]。但仍有10％～20％的患者移植后发生血液学复发且预后较差[5]。本组t(8;21) AML患者allo-HSCT后3年CIR达15.7％（95％*CI* 7.3％～26.8％），10例患者复发，在随访期间内最终有8例死亡。因此，区分allo-HSCT后t(8;21) AML高危复发人群，及时干预，避免复发，对改善生存显得尤为重要。

微小残留病（MRD）的检测是评价白血病预后和疗效的重要指标[8]–[9]。随着PCR技术的成熟，以RUNX1-RUNX1T1融合基因为基础的MRD动态检测，是有效预测t(8;21) AML复发的重要方法[5],[10]–[11]。但allo-HSCT后RUNX1-RUNX1T1融合基因动态检测时间及基因定量水平阈值尚不明确。有研究发现，移植后最初3个月内，RUNX1-RUNX1T1融合基因与初诊时基线水平（388％）相比，下降程度超过3个对数级的患者复发风险明显降低[5],[10]。本研究中，移植后1～3个月内融合基因下降水平≥3个对数级者3年CIR明显下降，同时我们还发现对处于CR1/CR2期的t(8;21) AML患者而言，0 d融合基因定量超过1.58％是allo-HSCT后复发的独立危险因素［*HR*＝28.849（95％*CI* 2.680～310.524），*P*＝0.006］。进一步分析发现，0 d融合基因高定量（≥1.58％）者，在移植后6个月内发生分子生物学阳性的事件增加（17/23）。由于融合基因定量水平的增加往往提示疾病复发[12]，这可以部分解释0 d融合基因高定量患者复发风险高的原因。由于0 d融合基因对复发有预测意义，因此对于0 d融合基因高定量患者，适当缩短RUNX1-RUNX1T1融合基因检测间隔时间可能有助于早期发现复发。

本研究还发现，初诊时骨髓流式细胞术原始细胞比例≥60％也是复发的独立危险因素［*HR*＝6.640（95％*CI* 1.448～30.457），*P*＝0.015］。但是形态学骨髓原始细胞比例≥60％与<60％两组移植后3年CIR差异无统计学意义，与文献[3]结果相一致。以往研究显示，CD56阳性t(8;21) AML患者CR后复发风险增加[13]–[14]，是预后不良的危险因素。因此骨髓原始细胞中表达与预后不良有关的表面分子的比例是否与复发有关，值得进一步探究。

以往研究发现，t(8;21) AML伴c-Kit基因突变患者的复发风险增加[15]–[16]。而且，c-Kit基因突变被认为是t(8;21) AML患者allo-HSCT后复发的高危因素[10]。但我们的研究发现，c-Kit基因突变组与野生组间，allo-HSCT后3年CIR差异无明显统计学意义（*P*＝0.877）。进一步分析发现，55例行基因检测的患者中，25例有c-Kit基因突变，其中11例0 d融合基因定量超过1.58％，30例无基因突变患者中，10例出现0 d融合基因定量超过1.58％，两者之间差异无统计学意义（*χ*^2^＝0.657，*P*＝0.580），这可能与c-Kit基因突变与未突变组间化疗强度的差异有关。此外，研究发现c-Kit基因不同突变位点间，在预测疾病复发方面存在差异[17]。但由于本研究中进行基因检测的样本数有限，因此需要纳入更多样本，进一步探究c-Kit基因突变以及其他基因突变对allo-HSCT后t(8;21) AML复发风险的影响。

除此之外，单倍型造血干细胞移植被认为有更强的抗白血病效应，可减少移植前MRD阳性AML复发的风险[18]。在本研究中，单倍型造血干细胞移植与同胞全合造血干细胞移植两组间复发风险差异无统计学意义（*P*＝0.669），有待进一步研究。

基于多因素分析结果，我们根据移植后复发的危险因素（初诊时骨髓流式细胞术原始细胞比例≥60％、0 d融合基因高定量）分成高危（具备2个危险因素）、中危（具备1个危险因素）、低危组（不具备危险因素），移植后3年CIR分别为57.1％、14.8％、3.0％（*P*<0.001）。对于行allo-HSCT的t(8;21) AML患者进行复发危险度分层，对高危复发患者及时给予供者淋巴细胞输注（DLI）等治疗，可能是减少复发、提高生存率的重要措施[10],[19]。

本研究结果显示，血液学复发是t(8;21) AML患者allo-HSCT后的主要死亡原因，0 d融合基因定量≥1.58％和初诊骨髓流式细胞术原始细胞比例≥60％是allo-HSCT后复发的独立危险因素。对高危复发患者加强MRD检测、及时抢先治疗可能有助于改善移植预后。

## References

[1] 主 鸿鹄, 黄 晓军 (2017). 我如何治疗t(8;21)急性髓系白血病[J]. 中华血液学杂志.

[2] Döhner H, Estey EH, Amadori S (2010). Diagnosis and management of acute myeloid leukemia in adults: recommendations from an international expert panel, on behalf of the European LeukemiaNet[J]. Blood.

[3] Zhu HH, Zhang XH, Qin YZ (2013). MRD-directed risk stratification treatment may improve outcomes of t(8;21) AML in the first complete remission: results from the AML05 multicenter trial[J]. Blood.

[4] Yoon JH, Kim HJ, Kim JW (2014). Identification of molecular and cytogenetic risk factors for unfavorable core-binding factor-positive adult AML with post-remission treatment outcome analysis including transplantation[J]. Bone marrow transplant.

[5] Wang Y, Wu DP, Liu QF (2014). In adults with t(8;21)AML, posttransplant RUNX1/RUNX1T1-based MRD monitoring, rather than c-KIT mutations, allows further risk stratification[J]. Blood.

[6] Yin JAL, O'Brien MA, Hills RK (2012). Minimal residual disease monitoring by quantitative RT-PCR in core binding factor AML allows risk stratification and predicts relapse: results of the United Kingdom MRC AML-15 trial[J]. Blood.

[7] Byrd JC, Dodge RK, Carroll A (1999). Patients with t(8;21)(q22;q22) and acute myeloid leukemia have superior failure-free and overall survival when repetitive cycles of high-dose cytarabine are administered[J]. J Clin Oncol.

[8] Pui CH, Pei D, Coustan-Smith E (2015). Clinical utility of sequential minimal residual disease measurements in the context of risk-based therapy in childhood acute lymphoblastic leukaemia: a prospective study[J]. Lancet Oncol.

[9] Jourdan E, Boissel N, Chevret S (2013). Prospective evaluation of gene mutations and minimal residual disease in patients with core binding factor acute myeloid leukemia[J]. Blood.

[10] Qin YZ, Wang Y, Xu LP (2017). The dynamics of RUNX1-RUNX1T1 transcript levels after allogeneic hematopoietic stem cell transplantation predict relapse in patients with t(8;21) acute myeloid leukemia[J]. J Hematol Oncol.

[11] Rücker FG, Agrawal M, Corbacioglu A (2019). Measurable residual disease monitoring in acute myeloid leukemia with t(8;21)(q22;q22.1): results from the AML Study Group[J]. Blood.

[12] Willekens C, Blanchet O, Renneville A (2016). Prospective long-term minimal residual disease monitoring using RQ-PCR in RUNX1-RUNX1T1-positive acute myeloid leukemia: results of the French CBF-2006 trial[J]. Haematologica.

[13] 刘 莎, 魏 旭东, 米 瑞华 (2015). CD56在t(8;21)成人急性髓系白血病患者中的表达及其与预后的相关性分析[J]. 中华血液学杂志.

[14] Baer MR, Stewart CC, Lawrence D (1997). Expression of the neural cell adhesion molecule CD56 is associated with short remission duration and survival in acute myeloid leukemia with t(8;21)(q22;q22)[J]. Blood.

[15] Duployez N, Marceau-Renaut A, Boissel N (2016). Comprehensive mutational profiling of core binding factor acute myeloid leukemia[J]. Blood.

[16] Boissel N, Leroy H, Brethon B (2006). Incidence and prognostic impact of c-Kit, FLT3, and Ras gene mutations in core binding factor acute myeloid leukemia (CBF-AML)[J]. Leukemia.

[17] Qin YZ, Jiang Q, Wang Y (2021). The impact of the combination of KIT mutation and minimal residual disease on outcome in t(8;21) acute myeloid leukemia[J]. Blood Cancer J.

[18] Chang YJ, Wang Y, Liu YR (2017). Haploidentical allograft is superior to matched sibling donor allograft in eradicating pre-transplantation minimal residual disease of AML patients as determined by multiparameter flow cytometry: a retrospective and prospective analysis[J]. J Hematol Oncol.

[19] Rettig AR, Ihorst G, Bertz H (2021). Donor lymphocyte infusions after first allogeneic hematopoietic stem-cell transplantation in adults with acute myeloid leukemia: a single-center landmark analysis[J]. Ann Hematol.

